# Supplementary material for: Differential LysoTracker Uptake Defines Two Populations of Distal Epithelial Cells in Idiopathic Pulmonary Fibrosis
Source: Cells. 2022 Jan 11;11(2):235. doi: 10.3390/cells11020235 (PMC8773634; doi:10.3390/cells11020235)
Supplement: Supplementary file 1 [file cells-11-00235-s001.zip › cells-1511858-supplementary.pdf]

Supplemental Material un Methods

Mouse lung tissue dissociation:

Saline perfused lungs were removed from the thoracic cavity, and 1ml RPMI solution containing 10% Dispase and 30µg/µl DNase I was instilled intra-tracheally and placed in a conical tube with 3 mls of the same dissociation solution. Following 30 minutes incubation in a 37°C water-bath, the lung lobes were isolated, minced and resuspended in 10ml RPMI 10% FBS 30ug/µl DNase I. The lung suspension was then pipetted repeatedly up and down until the individual lung pieces consolidated in one single piece of floating extracellular material and the solution became turbulent. The resulting cell suspension was filtered through 70-µm, 40-µm cell strainers. Cells were then spun down two times at 1200 RPM for 5 min, resuspended in FACS-buffer (HBSS 2%FBS 10µg /ml DNase I, 2mM HEPES, 1% Penicillin Streptomycin), and placed on ice in preparation for flow cytometry staining.

Human lung cell isolation

Subpleural lung tissue from explanted IPF lungs or excised from donor lungs due to donor-recipient size miss-match, was dissected and placed on ice. Tissue was minced with three scissors to pieces of approximately 1-2mm in diameter, washed three times with RPMI and incubated for 30 min and 37°C in RPMI containing Dispase (BD Cornig, 1:10 dilution) and 30µg/µl DNase I with continuous and gentle agitation on a rotating shaker. Tissue pieces were strained using first surgical gauze, then 100µm, 70µm and 40µm cell strainers and the strained solution was centrifuged and re-suspended in RPMI containing 10% FBS. Cells were washed two more time with RPMI 10% FBS, counted and shock-frozen in liquid nitrogen at a concentration of 2-10X10^{6} per vial until further use.

Flow cytometry analysis and FACS

All analyses were performed on Li-nitrogen stored cell preparation available through the European IPF registry biobank. Human lung cell suspension was thawed rapidly and cells were washed in 10 times the freezing volume of RPMI 10% FBS and maintained on ice throughout the procedure. Mouse cells were used immediately after isolation. Cells were resuspended in FACS buffer (HBSS, 2%FBS, 2 µg/µl DNase I, 2mM HEPES, 1% Penicillin Streptomycin) at a concentration of 10^{6} cells/100 µl and cell surface staining was performed using antibody master mixes whenever possible. Lysotracker incorporation assay (100nM solution) was performed for 30 minutes at room temperature in FACS buffer conform manufacturer's instructions. For intracellular staining, cell surface staining was first performed, then cells were fixed and permeabilized for 30 minutes at room temperature using the Foxp3 / Transcription Factor Staining Buffer Set according to the manufacturer instructions. Cells were washed following fixation and resuspended in 100µl of Permeabilization/wash buffer containing the primary antibody and incubated for one hour at room temperature,

washed three times, followed by Anti-rabbit IgG (H+L), F(ab')$_2$ Fragment (Alexa Fluor® 555 Conjugate Cell Signaling Technologies 1:1000).

The following controls were used: single color controls for instrument set-up, fluorescence-minus-one (FMO controls) for gating and no primary controls for indirect intracellular staining.

Data were acquired on a BD FACSCanto II (BD Biosciences) using BD FACSDiva software (BD Biosciences). Data were further analyzed using FlowJoX software (FlowJo, LLC).

For the cell sorting experiments, samples were thawed successively and stained as above. Cells were sorted through a 100uM nozzle in RPMI 10% and the sort purity was assessed for each sample. The cells were immediately centrifuged and resuspended in lysis buffer (RNA Mini kit) for RNA isolation. The entire procedure was performed at 4°C and cell lysates were stored at -80°C until RNA isolation was performed.

Immunofluorescence staining, imaging and analysis

Paraffin embedded human lung tissue was obtained from the European IPF registry biobank. For immunofluorescence staining, standard protocols were applied. Briefly, three-micron thick sections (3 μm) were cut from paraffin-embedded human lungs and mounted on positively charged glass slides (Super Frost Plus, Langenbrinck). Sections were deparaffinized in xylene and rehydrated in graded alcohol. Antigen retrieval was not necessary for the antibody stainings shown here. Lung sections were washed for 2 min in PBS, followed by blocking in PBS containing 5% (w/v) BSA, 2% (v/v) normal donkey serum (Jackson ImmunoResearch), and 0.1% Triton X-100 (Sigma) for 30 min. For immunofluorescence staining, sections were incubated overnight at 4°C with the appropriate antibodies (Supplemental table 2). Lung sections were then washed three times in PBS (5 min), followed by incubation with the appropriate fluorochrome-conjugated secondary antibodies (Supplemental table 2). Slides were washed one time in PBS (5 min) and were then incubated with Sudan Black (Sigma-Aldrich; 3% (w/v) in 70% ethanol) for 2 min. After extensive washing in PBS, slides were incubated with 4′,6-diamidino-2-phenylindole dihydrochloride (DAPI; Sigma-Aldrich) and mounted in Fluorescence Mounting Medium (Dako). As a negative control, the first antibody was omitted and the lung tissue slides were incubated only with fluorochrome-conjugated secondary antibodies. The mSP-B and proSP-B antibody staining required additional controls to ensure signal specificity and lack of secondary cross-reactivity: (1) no mSP-B primary antibody but amplified secondary, stripping in Na-Citrate and proSP-B primary and secondary staining; (2) amplified mSP-B, stripping, followed by omission of the proSP-B antibody; (3) mSP-B unamplified, no stripping followed by the proSP-B primary and secondary staining; (4) no mSP-B staining, only proSP-B primary/secondary antibody staining.

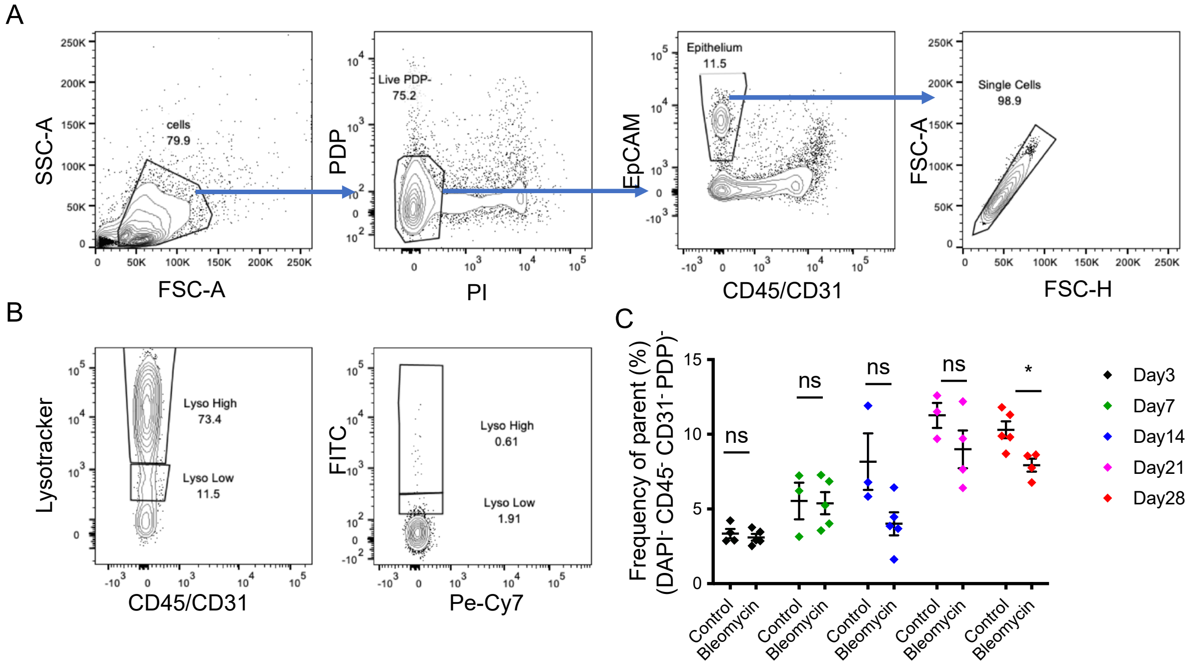

Figure S1. Characterization of lung cells in bleomycin model of lung fibrosis and human IPF lung (A) Representative panels showing the gating strategy of dissociated mouse lung: cell-debris discriminated based on size and granularity ("cells" gate); elimination of PDP+ and PI+ cells ("live PDP- " gate); identification of epithelial cells (EpCAM+ CD45- CD31-); doublet exclusion in the epithelial population ("single cell" gate). (B) Flow cytometry panels of Lysotracker incorporation in a control mouse lung (left) and the corresponding fluorescence minus one (FMO) gating control. (C) Statistical analysis of the epithelial compartment (PI- CD45- CD31- PDP- EpCAM+)

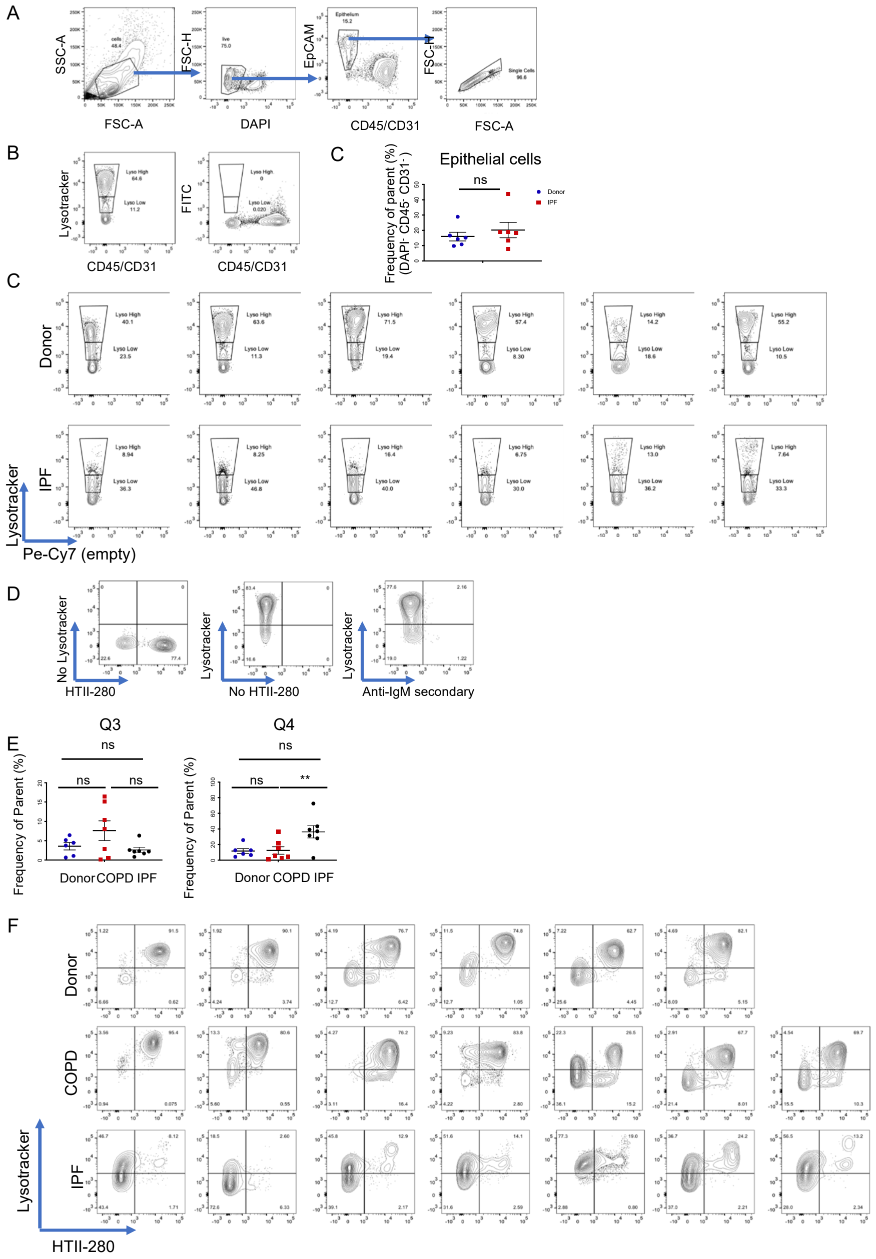

Figure S2. Lysotracker uptake in the epithelial compartment of the human lung. (A) Representative panels showing the gating strategy of dissociated human lung: cell-debris discriminated based on size and granularity ("cells" gate); elimination of DAPI+ cells ("live" gate); identification of epithelial cells (EpCAM+ CD45- CD31-); doublet exclusion in the epithelial population ("single cell" gate). (B) Flow cytometry panels of Lysotracker incorporation in a control human lung (left) and the corresponding fluorescence minus one (FMO) gating control. Statistical analysis of the proportion of epithelial cells (DAPI- CD45- CD31- EpCAM+) in donor and IPF samples. Data are presented as the mean ± SEM. *p < 0.05, **p < 0.01, ***p < 0.001, ns = not significant by Student t-test. (C) Individual panels of Lysotracker incorporation in the epithelial compartment of each donor and IPF patient. (D) Gating controls for the Lysotracker / HTII-280 analysis. First two panels correspond to the respective FMO controls for Lysotracker and HTII-280, the third panel shows the background of the secondary antibody used to detect HTII-280. (E) Statistical analysis of the proportion of Q3 and Q4 populations in donor, COPD and IPF patients. Data are presented as the mean ± SEM of Log10 (MFI). Statistical analysis was performed on Log(10) values. **p<0.01, n.s. = not significant by ANOVA. (F) Individual panels of Lysotracker/HTII-280 distribution in the epithelial compartment of each donor, COPD and IPF patient.

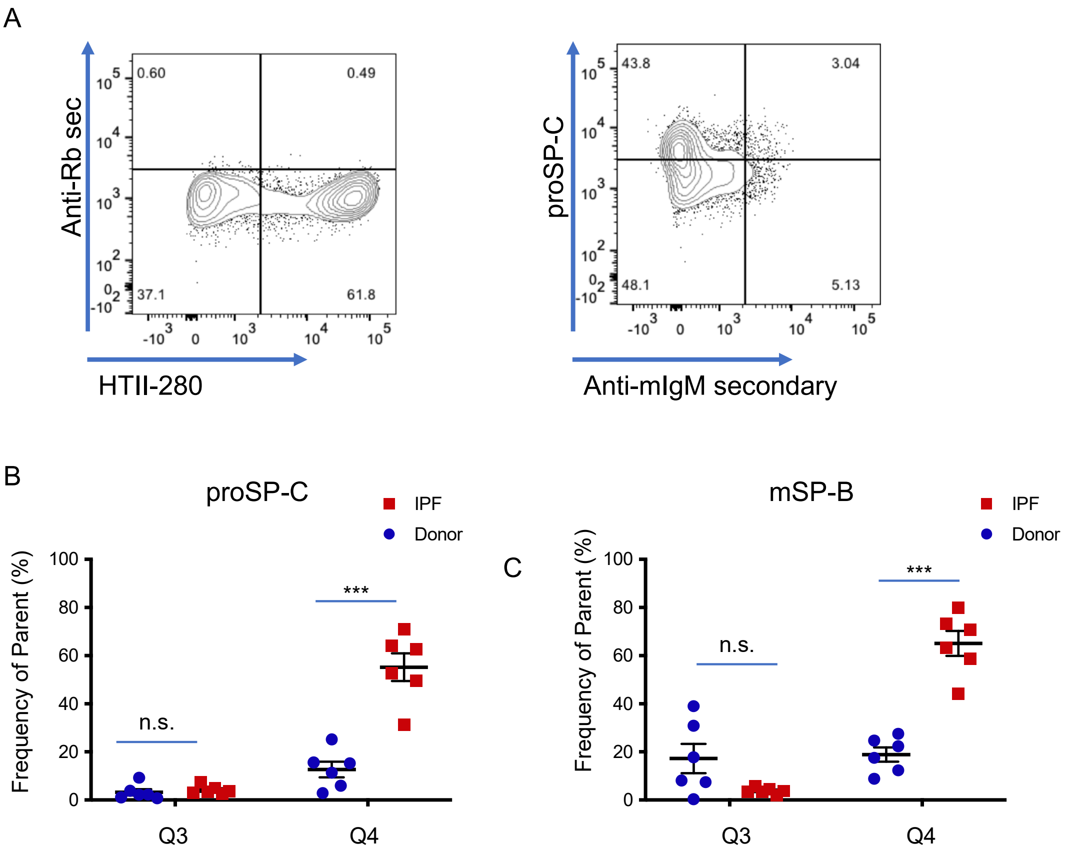

Figure S3. Surfactant protein expression in the epithelial compartment of donor and IPF lung. (A) Staining controls used to define the gating of the proSP-C and mSP-B vs HTII-280 analysis. First panel shows the "no primary" FMO control which is shared by the proSP-C and mSP-B, second panel shows the "no primary" FMO control for HTII-280. (B) Quantification of the population frequency of Q3 and Q4 in proSP-C stained donor and IPF lung samples shown in Figure 3A. (C) Quantification of the population frequency of Q3 and Q4 in mSP-B stained donor and IPF lung samples shown in Figure 3E.

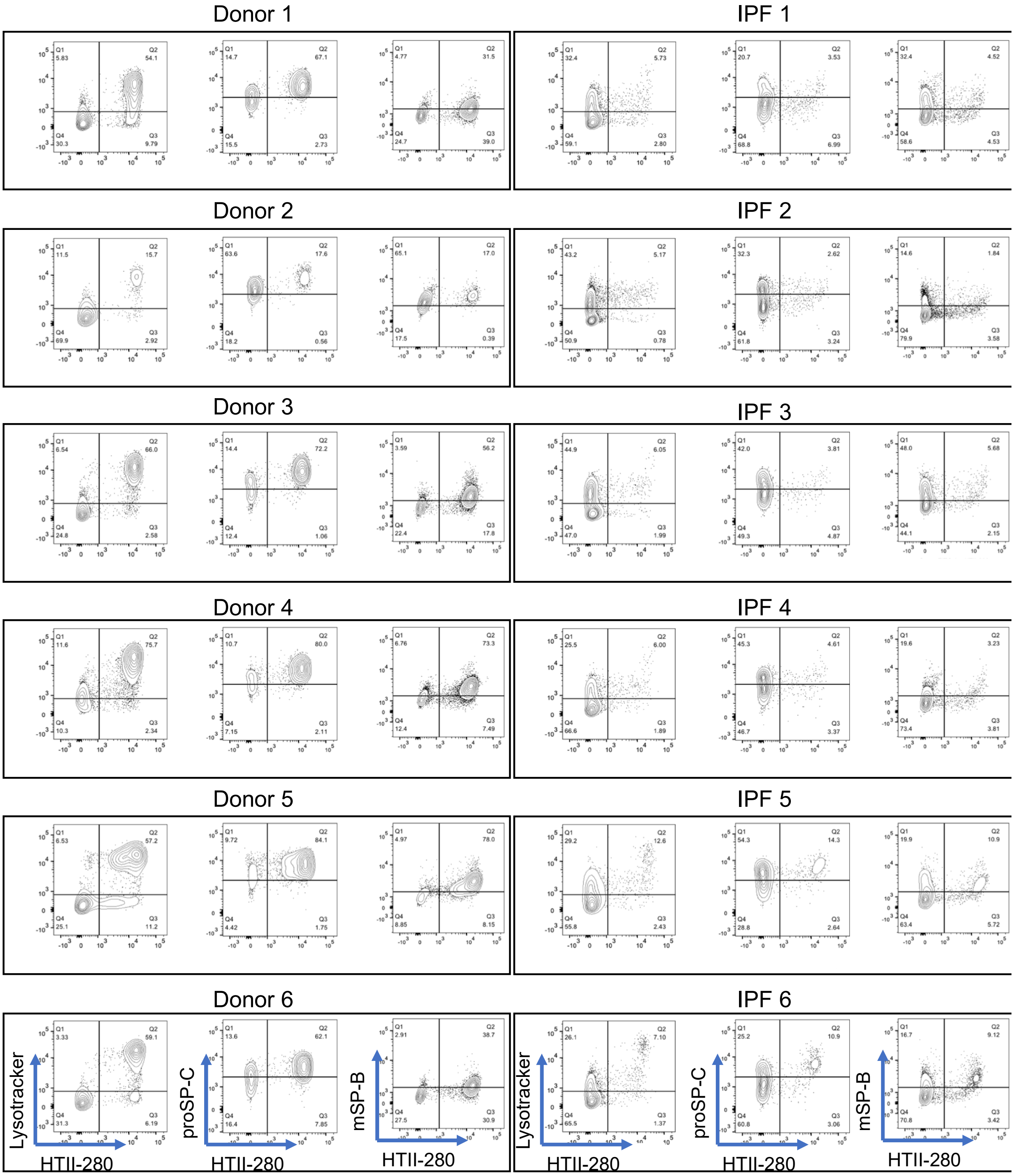

Figure S4. Individual flow cytometry panels of Lysotracker, proSP-C and mSP-B vs HTII-280 expression in the epithelial compartment (DAPI⁻ CD45⁻ CD31⁻ EpCAM⁺) of all donor (left column, n=6) and IPF (right column, n=6) lung preparations shown in Figure 4.

A

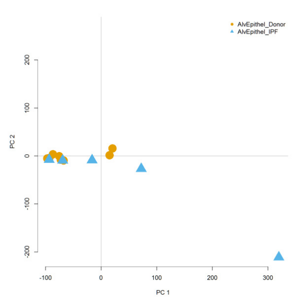

B

Gene	Coefficient	Significance (-lo	Donor	IPF
KRT5	8.56	8.61	3.39	11.96
KRT6A	6.89	5.16	3.90	10.79
UGT1A6	6.75	4.77	4.25	11.00
TRIM29	6.19	5.27	5.25	11.44
MMP10	6.14	3.95	3.10	9.24
CRLF1	6.12	6.38	4.79	10.90
KDR	5.64	4.10	3.39	9.02
KRT17	5.58	5.41	4.20	9.78
MXRA5	5.38	5.58	3.57	8.95
SIX1	5.23	4.23	6.16	11.40
KRT14	5.07	6.81	5.19	10.25
SPRR1B	5.04	4.15	3.80	8.83
FHL2	5.03	2.65	4.18	9.21
CLCA2	5.02	3.96	3.64	8.66
SPAG6	5.00	3.60	5.66	10.66
MMP7	4.96	2.78	6.77	11.73
ITGB4	4.96	3.46	4.24	9.21
DNAH2	4.95	3.92	4.01	8.96
S100A2	4.92	7.03	9.97	14.88
BPIFB1	4.90	6.14	6.65	11.55

Gene	Coefficient	Significanc	Donor	IPF
LPL	-6.15	7.33	10.11	3.96
BMP1	-5.72	7.39	8.84	3.13
TWIST2	-5.62	4.89	12.09	6.47
COLEC12	-5.27	5.59	9.29	4.02
CCDC141	-5.24	5.82	9.62	4.38
FADS1	-5.09	4.60	11.78	6.69
TTN	-5.00	5.00	10.43	5.42
CHI3L2	-4.99	4.83	10.23	5.24
AATK	-4.88	2.75	11.03	6.14
ITPRIPL2	-4.79	2.77	11.16	6.36
KCNA3	-4.77	2.77	8.94	4.17
PLA2G1B	-4.57	3.10	13.22	8.66
GLS	-4.54	3.15	10.77	6.23
PHB2	-4.39	2.85	10.15	5.76
SF3B3	-4.37	2.82	9.88	5.51
DCTN4	-4.35	3.47	9.83	5.48
CNTROB	-4.29	2.54	9.39	5.10
MT1E	-4.28	5.31	7.67	3.39
CSF3R	-4.25	3.97	12.18	7.93
SMIM11	-4.25	3.48	9.44	5.19
TMEM97	-4.21	2.79	11.86	7.65
ABCA3	-4.18	3.53	12.61	8.44

C

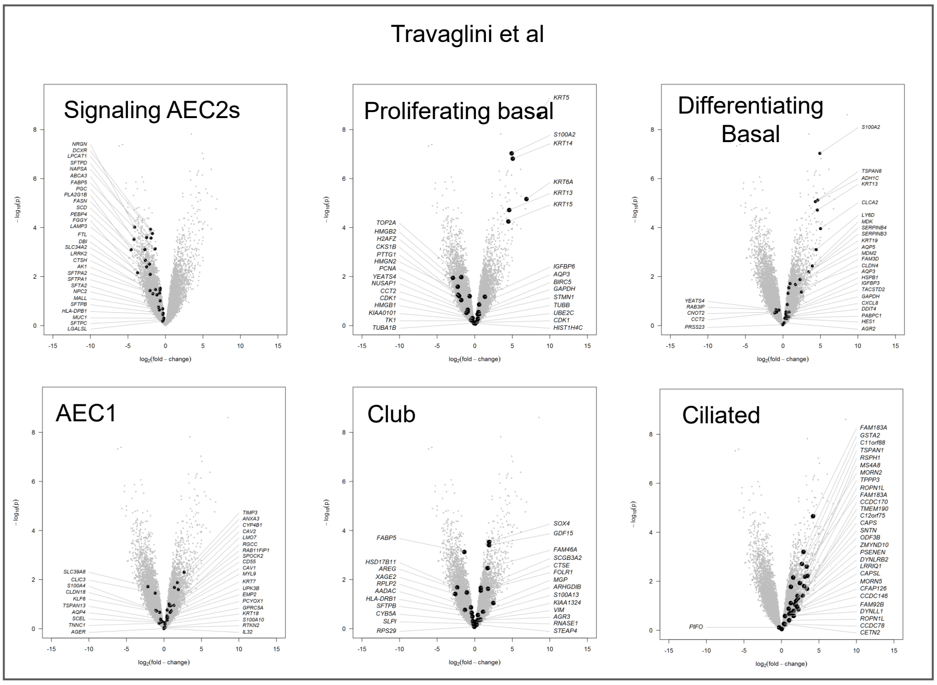

D

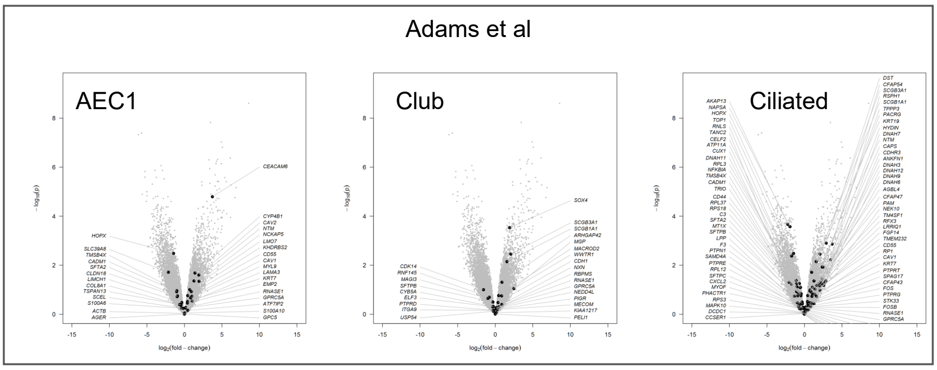

Figure S5. Transcriptomic profiling of the Lyso^{pos} population in IPF. (A) PCA analysis of the IPF Lyso^{pos} (blue triangles) and Donor Lyso^{pos}(orange circles). (B) List of the 20 most up-regulated (left)and down-regulated (right) genes in IPF Lyso^{pos} vs donor Lyso^{pos} population. (C), (D) The transcriptomic signatures of AEC2 identified by Travaglini et al. and Adams et al. were superimposed on the vulcano plots depiction of the up and downregulated genes in IPF Lyso^{pos} compared to donor Lyso^{pos} . (C)Transcriptomic signatures of different epithelial cell populations in donor lung identified by Travaglini et al transposed onto our Do/IPF data. (D) Transcriptomic signatures of different epithelial cell populations in IPF lung identified by Adams et al transposed onto our Do/IPF data.

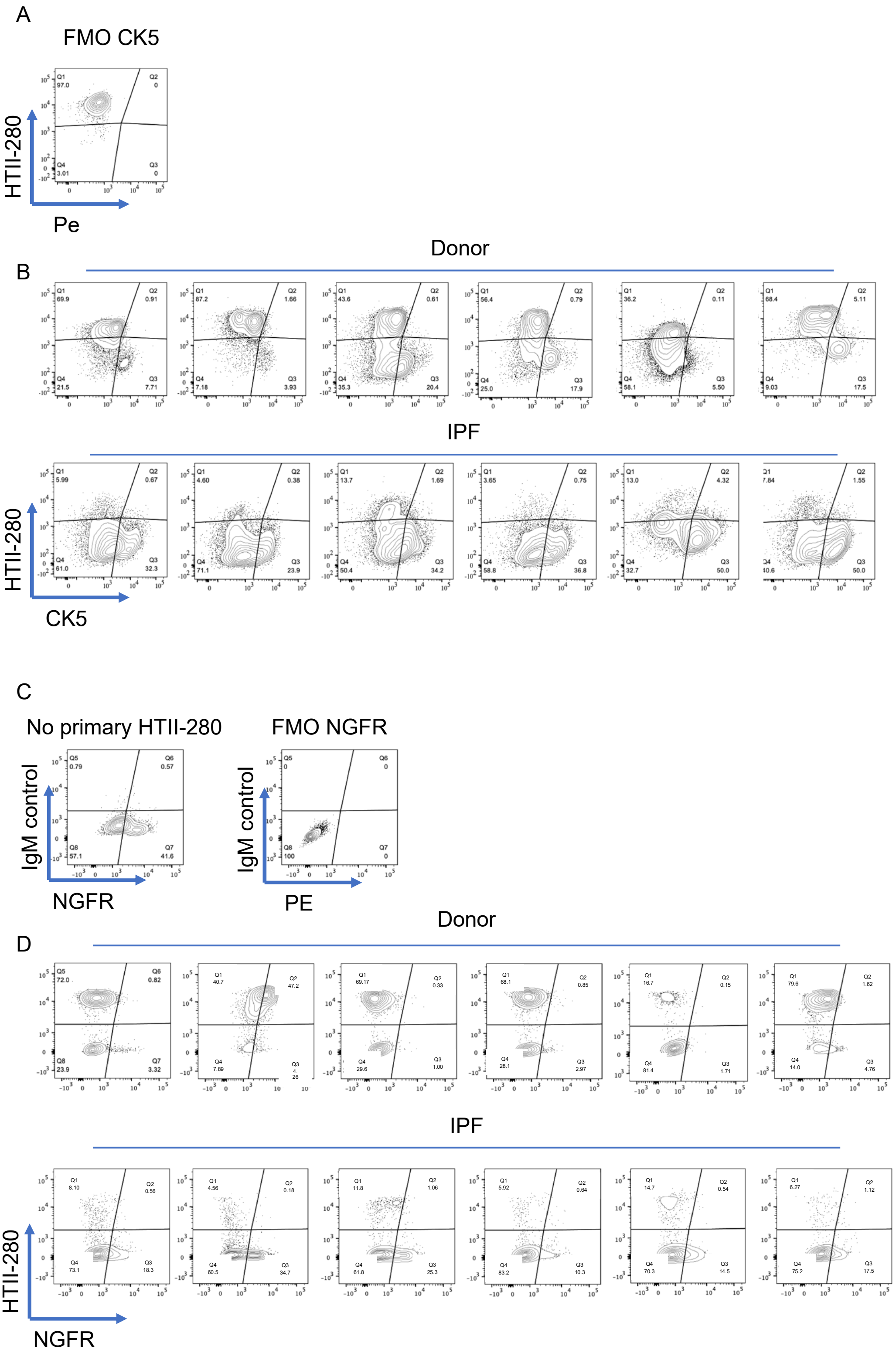

Figure S6. CK5 and NGFR expression in donor and IPF epithelial cells. (A) FMO gating control for CK5. (B) CK5 HTII-280 flow cytometry plots for individual donor and IPF patients shown in Figure 6. (C) No primary HTII-280 and NGFR FMO controls. (D) NGFR HTII-280 flow cytometry plots for individual donor and IPF patients shown in Figure 6.

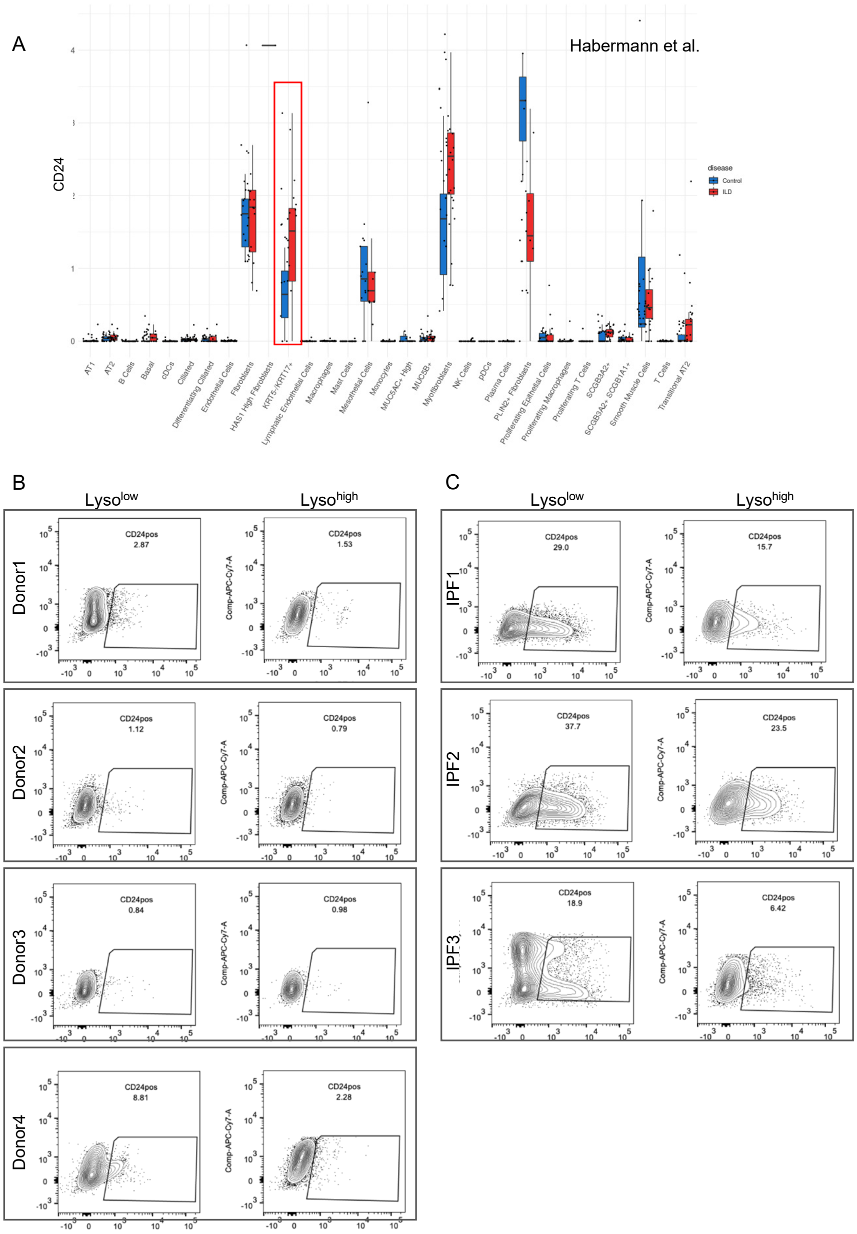

Figure S7. CD24 expression in donor and IPF lung. (A) Differential expression of CD24 in donor and IPF scNGS data published by Habermann et al. (B), (C)Individual flow cytometry panels of n=4 donor (B) and n=3 IPF (C) of Lysotracker incorporation in the epithelial cell compartment (left panel) shown in Figure 7.

Table S1. Important materials

Materials	Company	Cat#
Bleomycinsufate, injection solution 15,000 intl. units	Hexal	
Fetal bovine serum (FBS)	Biochrom	S0615
Bovine serum albumin (BSA) Fraction V	Roth	8076.3
Normal Donkey serum	Jackson Immuno Research	017-000-001
Penicillin/Streptomycin	Gibco	15140-122
Dispase	Corning	354235
DNAseI	Sigma-Aldrich	DN25-1G
Triton X-100	Sigma Aldrich	T8787
Sudan black	Sigmal-Aldrich	199664-25G
Fluorescence Mounting Medium	DakoCytomation	S3023
Lysotracker Green	Thermo Fisher Scientific	L7526
HBSS	Sigma-Aldrich	55037C-1000M
RPMI 1640 Media	Gibco	61870036
DAPI	Sigma-Aldrich	D9542
RNeasy kit (total RNA isolation)	Qiagen	74104
Alexa Fluor488 Tyramide SuperBoost Kit, goat	Thermo Fisher Scientific	B40922
eBioscience™ Foxp3 /Transcription Factor Staining Buffer Set	Thermo Fisher Scientific	00-5523-00
Adhesion Microscope Slides SuperFrost Plus®	Langenbrink	03-0060

Table S2. Antibodies used in experiments

**Primary antibody target**	**Company**	**Cat no**	**ICH/ICC/IF**	**FACS (per 10*6cells)**
anti human proSP-B	Millipore	AB3430		1_200
anti human mature SP-B	Seven Hills Bioreagents	WRAB-48604	1_2000	1_200
anti human Cytokeratin 5	Biolegend	905901	1_20000	1_500
anti human Cytokeratin 5 AlexaFluor 488	Abcam	ab193894		1_500
anti human proSP-C	Millipore	AB3786	1_3000	1_200
anti human NGFR (p75) APC	Biolegend	345108	0.25_100	5_100
anti human EpCAM APC-Cy7	Biolegend	324222		0.5_100
anti human CD45 Pe-Cy7	Biolegend	304016		2.5_100
anti human CD31 Pe-Cy7	Biolegend	303120		0.5_100
anti human HTII-280	TerraceBiotech	TB-27AHT2-280		1_500
anti human CD24 PE	Biolegend	983602		2_100
anti mouse EpCAM Pe-Cy7	Biolegend	118216		0.25_100
anti mouse CD45 APC-Cy7	Biolegend	103114		0.25_100
anti mouse CD31 APC-Cy7	Biolegend	102418		0.25_100
**Secondary antibodies:**				
Donkey anti-rabbit Alexa-Fluor 488	ThermoFisherScientific	A21206	1_500	1_500
Donkey anti-rabbit Alexa-Fluor 555	ThermoFisherScientific	A31572	1_500	1_500
Donkey anti-rabbit Alexa-Fluor 647	ThermoFisherScientific	A31573	1_500	1_500
Donkey anti-mouse Alexa-Fluor 488	ThermoFisherScientific	A21202	1_500	1_500
Donkey anti-mouse Alexa-Fluor 555	ThermoFisherScientific	A31570	1_500	1_500
Donkey anti-mouse Alexa-Fluor 647	ThermoFisherScientific	A31571	1_500	1_500
Donkey anti-chicken Alexa-Fluor 488	ThermoFisherScientific	A11039	1_500	1_500
Donkey anti-chicken Alexa-Fluor 555	ThermoFisherScientific	A21437	1_500	1_500
Anti-Mouse F(ab')2 Fragment Alexa Fluor 488	Cell SignalingTechnologies	CST4408	1_500	1_500
Anti-Mouse F(ab')2 Fragment Alexa Fluor 555	Cell SignalingTechnologies	CST4409	1_500	1_500
Anti-Rabbit F(ab')2 Fragment Alexa Fluor 488	Cell SignalingTechnologies	CST4412	1_500	1_500
Anti-Rabbit F(ab')2 Fragment Alexa Fluor 555	Cell SignalingTechnologies	CST4413	1_500	1_500
Goat anti-Mouse IgM Alexa Fluor 488	ThermoFisherScientific	A-21042	1_1000	1_1000
Goat anti-Mouse Alexa Fluor 488	ThermoFisherScientific	A-11001	1_1000	1_1000
